# Addition of probiotics to antibiotics improves the clinical course of pneumonia in young people without comorbidities: a randomized controlled trial

**DOI:** 10.1038/s41598-020-79630-2

**Published:** 2021-01-13

**Authors:** Chang Hun Lee, Yunjung Choi, Seung Young Seo, Seong-Hun Kim, In Hee Kim, Sang Wook Kim, Soo Teik Lee, Seung Ok Lee

**Affiliations:** 1 Department of Internal Medicine, Naval Pohang Hospital, Pohang, South Korea; 2 Division of Gastroenterology, Department of Internal Medicine, Jeonbuk National University Medical School, Research Institute of Clinical Medicine of Jeonbuk National University-Biomedical Research Institute of Jeonbuk National University Hospital, Jeonju, South Korea; 3 Division of Rheumatology, Department of Internal Medicine, Jeonbuk National University Medical School, Research Institute of Clinical Medicine of Jeonbuk National University-Biomedical Research Institute of Jeonbuk National University Hospital, Jeonju, South Korea

**Keywords:** Clinical microbiology, Randomized controlled trials

## Abstract

This study was aimed at investigating the clinical efficacy of probiotics in pneumonia patients. To this end, we enrolled 80 participants diagnosed with pneumonia at Naval Pohang Hospital, Pohang, Korea, from May 2016 to January 2017. The participants were randomly assigned to the control and probiotic groups depending on whether they received probiotics. All participants clinically improved but 22.6% of the participants complained of abnormal stool habits after pneumonia treatment. In comparison, fever duration was significantly shorter in the probiotic group, and the group exhibited an improved general condition. The probiotic group also showed better stool characteristics according to the Bristol stool scale (P = 0.009). Notably, the serum hs-CRP levels were significantly lower in the probiotic group at 2 weeks of treatment (*P* = 0.015), and all participants in the probiotic group achieved their levels within the normal range. Flow cytometry was used to analyze T-helper 17 (Th17) cells and regulatory T cells (Tregs). Tregs were promoted and the Th17 cell/Treg ratio was suppressed after 2 weeks of treatment in the probiotic group (*P* = 0.007 and 0.037, respectively). This study demonstrated that probiotics improved clinical symptoms and normalized inflammatory biomarker levels in patients with pneumonia. Early infection and inflammation recovery may be due to the immunomodulatory effects of probiotics by facilitating the subset of Tregs and suppressing the Th17 cell/Treg ratio.

## Introduction

Probiotics are microorganisms that have beneficial effects on the human body. According to the definition of the Food and Agriculture Organization of the United Nations/World Health Organization (FAO/WHO), probiotics are live microorganisms that confer a health benefit to the host when administered in adequate amounts^1,2^. Many probiotics are originally isolated from the gastrointestinal tract and are associated with gut microbiota^3^. Previous studies have shown that probiotics can prove beneficial in improving gastrointestinal (GI) tract health, modulating the immune system, and relieving allergies or autoimmune diseases. Most mechanisms by which probiotics exhibit their effects are unknown but may include antagonistic effects against pathogens and stimulation of immunomodulatory cells^4^.

Currently, the clinical applications of probiotics are mainly related to the improvement of GI symptoms. According to the World Gastroenterology Organization Global Guidelines updated in 2017, administration of certain probiotic strains for the management of antibiotic- and *Clostridium difficile*-associated diarrhea, hepatic encephalopathy, pouchitis, and lactose intolerance are considered “evidence-based”^1^. The typical effects of probiotics in clinical settings are improvements in fecal consistency and GI symptoms^5^. Several randomized controlled trials and meta-analyses have shown that the use of probiotics has a beneficial effect in the aforementioned environment^6–10^. Nevertheless, further evaluations are necessary to establish the benefits of probiotics in other fields^11^.

A previous study have shown that probiotics may also play a role in ameliorating secondary infections in H7N9 virus-infected patients^12^. Another study has shown the effectiveness of probiotics for the prevention of ventilator-associated pneumonia (VAP)^13^. Data from clinical trials and meta-analyses also demonstrated shorter treatment durations and diminished inflammation in clinical settings in which probiotics were used to manage respiratory tract infections^14–16^. However, most studies focused on the preventive role of probiotics in respiratory infections and the subjects of many studies were children. Moreover, some studies have revealed conflicting results and the underlying mechanisms are not well elucidated.

With regard to immunologic responses, if probiotic administration has immune-boosting effects, it may help clear pathogens; in turn, an enhanced immune reaction could result in severe tissue damage^17^. CD4^+^ T cells, which are a major T-cell subset, play a central role in immune system functions against bacterial, viral, and fungal pathogens. Specific subpopulations of T cells, typically identified as CD4^+^ CD25^+^ Foxp3^+^ T cells, are known to dampen immune responses^18^. Regulatory T cell (Treg) activity can benefit the host by minimizing damage to the lung tissue. On the other hand, it may limit the magnitude of effector responses, which may result in inadequate infection control. T-helper 17 (Th17) cells, which constitute a distinct subset of helper T cells with expression of the nuclear receptor RORγt, are known to play a crucial role in host defense against various pathogens. Although they are required for host defense against pathogens, they produce a large amount of inflammatory cytokines and potentially induce tissue damage^19^. Several studies have shown the alteration of Tregs and Th17 cells in infectious conditions^17,19^. Recently, an imbalance between Th17 cells and Tregs has been observed in various autoimmune diseases and infectious or inflammatory conditions^20–22^. Therefore, we planned to evaluate the efficacy of probiotic administration in pneumonia patients without underlying comorbidities and share similar demographic characteristics. Flow cytometry showed changes in specific T-cell subsets, i.e., Tregs and Th17 cells, during the treatment. We hypothesized that co-administration of probiotics may facilitate clinical improvement in patients with pneumonia by modulating immune responses through Tregs and Th17 cells.

## Results

### Demographic and baseline clinical characteristics of the patients

**Figure 1:**
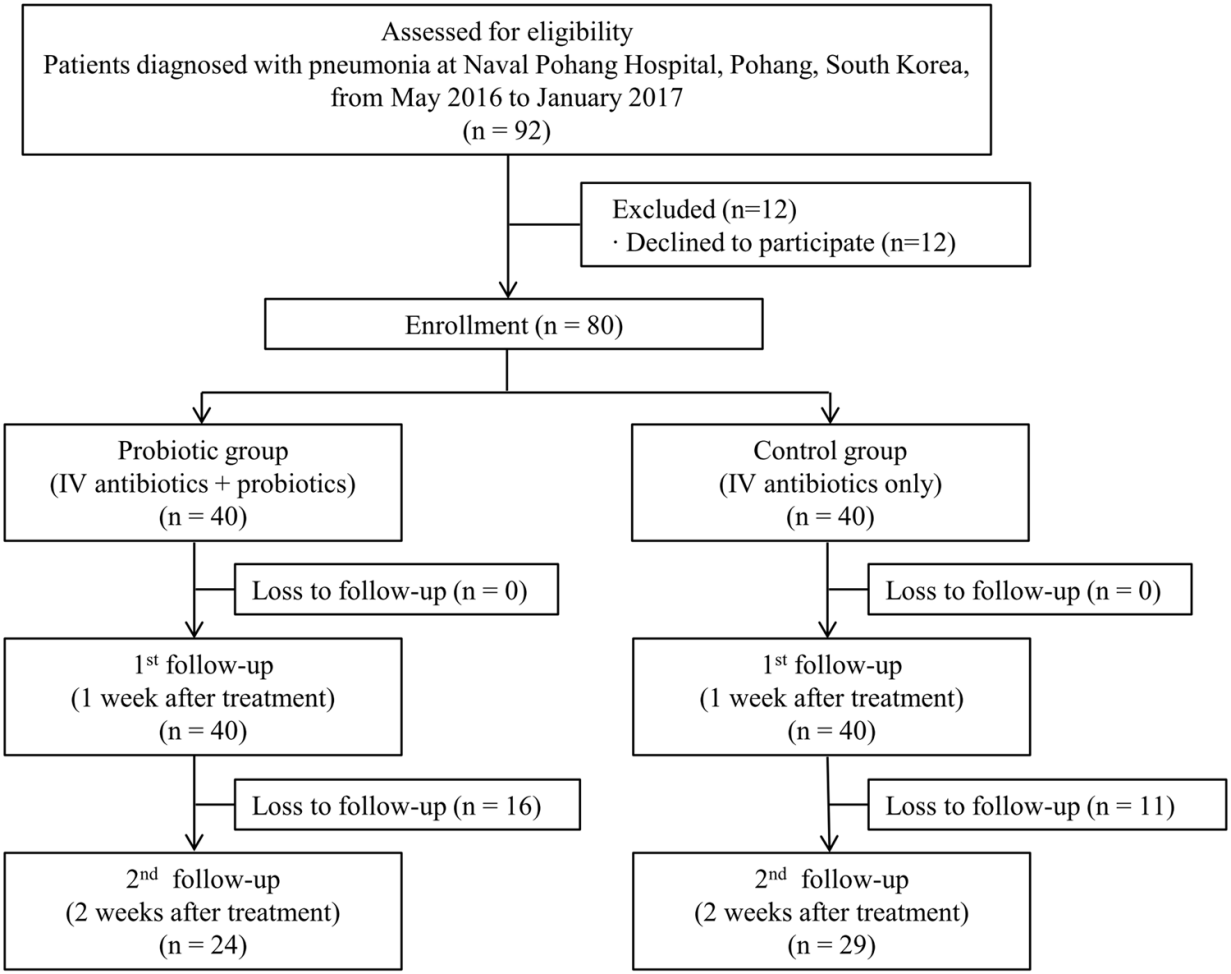
Flow chart of the study.

Figure 1 shows a flowchart of the enrolled subjects prepared according to the CONSORT 2010 form. The patients’ demographic and baseline characteristics are shown in Table 1. The average age of the subjects was 20.0 ± 1.1 years, and all participants were male. More than half of the subjects had a smoking history, which was 2.2 ± 2.2 pack-years. They did not have underlying diabetes, hypertension, chronic liver disease, or cancer. Among these subjects, five (6.2%) had a history of pneumonia treatment.

**Table 1:** Demographics and baseline clinical characteristics.

Characteristics	Control group (n = 40)	Probiotic group (n = 40)	*P* value
Age	19.9 ± 1.0	20.1 ± 1.2	0.416
Sex (male)	40 (100.0%)	40 (100.0%)	–
BMI, kg/m^2^	22.8 ± 4.4	23.4 ± 3.2	0.501
**Smoking history**	25 (62.5%)	21 (52.5%)	0.497
Pack-years	1.8 ± 1.5	2.7 ± 2.7	0.204
Medication history	0 (0.0%)	0 (0.0%)	–
Hypertension	0 (0.0%)	0 (0.0%)	–
Diabetes	0 (0.0%)	0 (0.0%)	–
Chronic liver disease	0 (0.0%)	0 (0.0%)	–
Malignancy	0 (0.0%)	0 (0.0%)	–
Prior pneumonia history	2 (5.0%)	3 (7.5%)	1.000
**Clinical manifestations**			
Fever	25 (62.5%)	26 (65.0%)	1.000
Chills	31 (77.5%)	32 (80.0%)	1.000
Headache	16 (40.0%)	15 (37.5%)	1.000
Cough	38 (95.0%)	40 (100.0%)	0.474
Coryza	26 (65.0%)	31 (77.5%)	0.323
Sputum	32 (80.0%)	38 (95.0%)	0.091
Sore throat	19 (47.5%)	19 (47.5%)	1.000
Chest pain	14 (35.0%)	21 (52.5%)	0.176
Abdominal pain	3 (7.5%)	1 (2.5%)	0.608
**Vital Sign**			
Systolic BP, mmHg	121.4 ± 11.1	120.2 ± 14.1	0.666
Diastolic BP, mmHg	73.6 ± 10.4	76.2 ± 11.6	0.296
HR, beat per min	80.1 ± 14.2	86.5 ± 15.5	0.058
RR, breath per min	16.1 ± 0.3	16.1 ± 0.8	0.474
Boby temperature	37.3 ± 0.8	37.5 ± 1.0	0.277
General condition (VAS)	4.5 ± 1.3	4.0 ± 1.2	0.058
**Bristol Stool Scale**			0.483
Type 1 or 2	1 (2.5%)	2 (5.0%)	
Type 3, 4, or 5	38 (95.0%)	35 (87.5%)	
Type 6 or 7	1 (2.5%)	3 (7.5%)	
**Laboratory parameters**			
WBC, /μl	8067.8 ± 5257.4	8218.5 ± 3556.7	0.881
Hemoglobin, g/dl	14.2 ± 1.3	14.5 ± 1.3	0.316
Platelet, /μl	209.7 ± 71.1	240.2 ± 98.3	0.116
PT (INR)	1.2 ± 0.1	1.2 ± 0.1	0.541
hs-CRP, mg/dl	68.7 ± 59.8	70.8 ± 53.2	0.869
ESR, mm/hr	13.2 ± 10.7	9.2 ± 4.3	0.485
ALP, IU/l	66.8 ± 21.7	65.0 ± 17.5	0.682
AST, IU/l	33.6 ± 18.9	40.5 ± 25.0	0.166
ALT, IU/l	28.3 ± 25.2	31.5 ± 19.4	0.523
Total bilirubin, mg/dl	0.5 ± 0.3	0.6 ± 0.4	0.115
Protein, g/dl	6.9 ± 0.4	7.0 ± 0.5	0.207
Albumin, g/dl	4.2 ± 0.4	4.2 ± 0.3	0.627
BUN, mg/dl	12.2 ± 2.5	11.9 ± 3.0	0.595
Creatinine, mg/dl	0.9 ± 0.1	0.9 ± 0.1	0.510
Sodium, mmol/l	135.5 ± 2.6	135.6 ± 2.6	0.822
Potassium, mmol/l	3.9 ± 0.6	4.1 ± 0.2	0.097
Chloride, mmol/l	100.3 ± 2.8	100.0 ± 2.4	0.682
LD, IU/l	231.7 ± 75.9	239.6 ± 56.7	0.620

BMI, body mass index; BP, blood pressure; HR, heart rate; RR, respiratory rate; VAS, visual analogue scale; WBC, white blood cells; PT, prothrombin time; INR, international normalized ratio; hs-CRP, high-sensitivity C-reactive protein; ESR, erythrocyte sedimentation rate; ALP, alkaline phosphatase; AST, aspartate aminotransferase; ALT, alanine aminotransferase; BUN, blood urea nitrogen; LD, lactate dehydrogenase.

### Results of microbial analyses

Laboratory test results demonstrating the etiology of pneumonia are shown in Supplementary Table S1. Sputum culture was positive for *Pseudomonas* and *Streptococcus* species in two participants. Blood culture was positive for methicillin-sensitive *Staphylococcus aureus* (MSSA) in one subject. Thirty-two subjects (41.0%) were positive for *Mycoplasma pneumoniae* IgM. Respiratory PCR analysis showed positive results for 24 (60.0%) and 23 (57.5%) subjects in the probiotic and control groups, respectively. The most common PCR-positive viruses were adenovirus and rhinovirus, for which 30 subjects (37.5%) tested positive. There were also respiratory syncytial virus (RSV), influenza, and parainfluenza PCR-positive cases.

### Follow-up clinical findings

After treatment, all participants showed clinical improvement, and there was no serious adverse event in either group during the study period. During the treatment period, the visual analog scale (VAS) scores for patients’ general condition increased from 4.2 to 7.3 and high-sensitivity C-reactive protein (hs-CRP) levels decreased from 69.8 ± 56.3 mg/dL to 3.0 ± 5.0 mg/dL. Stool characteristics of constipation or diarrheal type increased from 8.8% to 22.6%, and in subjects with alanine aminotransferase (ALT) elevation, they increased from 16.2% to 39.2%. The results are shown in Supplementary Fig. S1.

**Figure 2:**
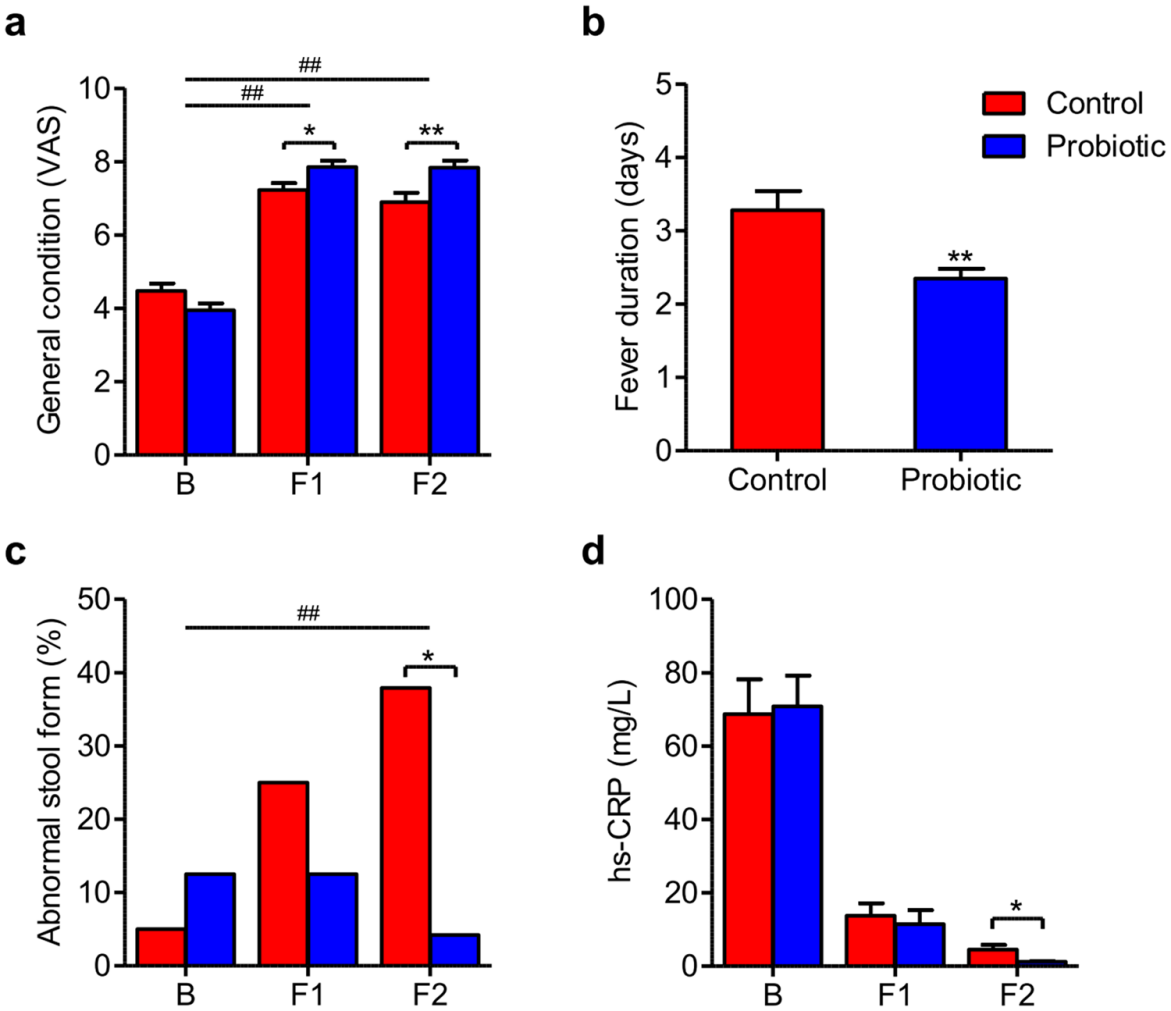
Comparison of clinical manifestations depending on the administration of the probiotic. Values are mean ± SEM. *P < 0.05, **P < 0.01 versus control, and ^##^*P* < 0.01 versus control comparing Δ-values. B, baseline; F1, 1 week after treatment; F2, 2 weeks after treatment.

Comparisons of clinical parameters between probiotic and control groups are shown in Fig. 2, Supplementary Tables S2, S3, and Fig. S2. Among the subjects who had a fever at the time of admission, the mean fever duration was significantly shorter in the probiotic group (2.3 ± 0.7 vs. 3.3 ± 1.3 days, P = 0.003). Furthermore, the VAS scores for patients’ general condition at 2 weeks of treatment were significantly higher among the probiotic group (7.8 ± 1.0 vs. 6.9 ± 1.4, P = 0.007). The improvement in the general conditions during the follow-up period was also significantly higher in the probiotic group. Regarding the stool patterns, the percentage of subjects with abnormal stool characteristics according to the Bristol stool scale was lower in the probiotic group (4.2% vs. 37.9%, P = 0.009). Notably, the serum hs-CRP levels were significantly lower in the probiotic group at 2 weeks of treatment, and all participants in the probiotic group had improved serum hs-CRP levels within the normal range (100.0% vs. 74.1%, P = 0.023). Meanwhile, there was no significant intergroup difference in the number of subjects with ALT. The between-group differences for the repeated measures on the general conditions or the serum hs-CRP levels during the enrolled period were not significantly different between the two groups.

### Flow cytometry analysis

**Figure 3:**
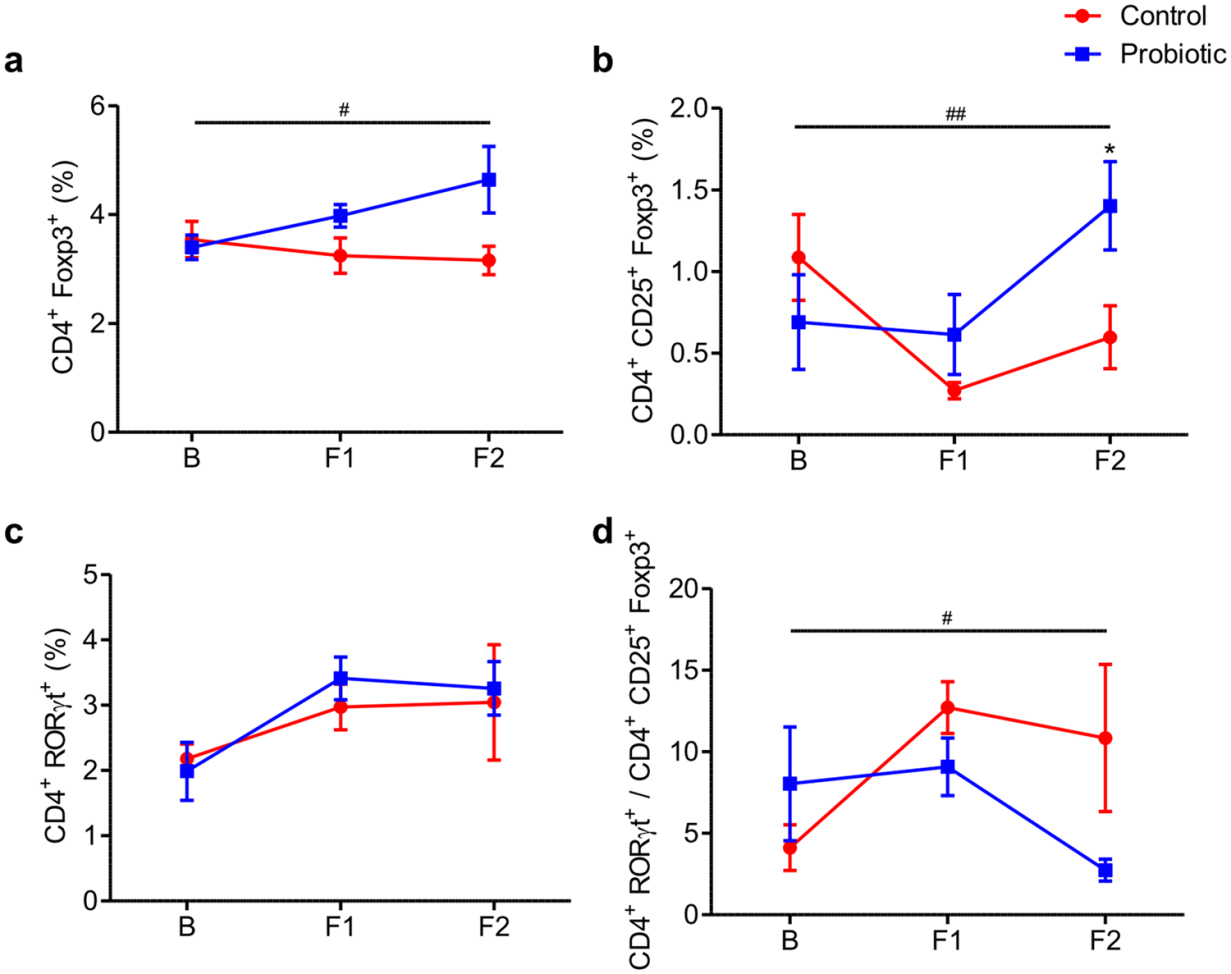
Flow cytometry analysis of T-cells (n = 4–8). Values are mean ± SEM. *P < 0.05 versus control, ^#^*P* < 0.05, and ^##^*P* < 0.01 versus control comparing Δ-values. B, baseline; F1, 1 week after treatment; F2, 2 weeks after treatment.

Four to eight samples were analyzed for each group, and the results of the flow cytometry analysis of the representative immune cells, Th17 cells and Tregs, are shown in Fig. 3. We verified the proportion of CD4 + CD25 + Foxp3 + Tregs and found that the cell population was significantly higher at 2 weeks of treatment in the probiotic group (P = 0.027). The increase in CD4^+^ Foxp3^+^ T cells and CD4^+^ CD25^+^ Foxp3^+^ Tregs at 2 weeks of treatment compared with baseline was also significantly higher in the probiotic group. Notably, the Th17 cell/Treg ratio was significantly suppressed in the probiotic group at 2 weeks of treatment (P = 0.037). On the other hand, the between-group differences for the repeated measures on CD4^+^ CD25^+^ Foxp3^+^ Tregs, CD4^+^ RORγt^+^ Th17 cells, or Th17 cell/Treg ratio during the follow-up period were not significantly different between the two groups.

## Discussion

Gut microbiota comprises tens of trillions of microorganisms, and their relationship to human health is being increasingly recognized. Gut commensals, although they can be regarded as pathogens immunologically, collaborate with the immune system and aid intestinal barrier function^23,24^. In this study, we evaluated the effect of probiotics in patients with pneumonia. The probiotic group showed rapid defervescence, better general condition, and early normalization of inflammation biomarkers as well as improved stool characteristics. The probiotic also had an immune-modulation effect, facilitating the subset of Tregs and suppressing the Th17 cell/Treg ratio.

Pneumonia is lung inflammation mainly caused by infections such as those with bacteria or viruses, which may progress to fatal conditions such as acute respiratory distress syndrome (ARDS) or sepsis. The outcome varies according to the underlying condition and variable factors. The main treatment for pneumonia is antibiotics, and β-lactam antibiotics and azithromycin are the standard empirical therapy for non-severe inpatient pneumonia^25,26^. It is believed that unique microbiota resides in the GI and respiratory tract mucosa and mediate host defense against pathogens^27^. Previous studies have shown some beneficial effects of certain strains of microbes on the recurrence of upper respiratory tract infections and nasal colonization^28^. Several studies have reported the preventive effect of probiotics against VAP in patients who received mechanical ventilation^13^. However, the level of evidence is still not satisfactory due to the small size of study or conflicting results; therefore, it is difficult to apply this evidence in the general management of pneumonia in clinical settings^29^.

Our data showed several beneficial effects of probiotics in pneumonia treatment. First, fever duration was shorter in the probiotic group. Fever is an important clinical parameter, probably caused by an underlying inflammatory condition, and normalization of body temperature is one of the first signs of clinical improvement. The participants’ general condition, as reflected by their VAS scores, improved early in the probiotic group. Furthermore, there were fewer complaints about symptoms, including improvements in bowel habits, which may influence the maintenance of better conditions during the treatment period. A large proportion of participants in our study had abnormal stool habits, which may be due to the infection itself or antibiotics associated. Diarrhea was the predominant type, and the number of subjects with abnormal stool patterns increased during the follow-up period. However, patients who were administered probiotics showed better stool consistency without abdominal symptoms. The percentage of subjects with abnormal stool patterns increased steadily in the control group, while that in the probiotic group decreased slightly, resulting in a statistically significant difference. Lastly, hs-CRP levels normalized, especially in the probiotic group at the end of treatment (at 2 weeks of treatment). The level of hs-CRP, which is induced by IL-6 and synthesized in the liver, is a commonly used inflammatory biomarker. These results show that probiotic use may facilitate clinical improvement in patients with pneumonia.

The concept of probiotics as immune modulators and their various applications for preventing, modifying, or treating diseases have been highlighted. Probiotics affect various organs outside the GI tract, and gut-lung crosstalk may be involved in the probiotic-mediated control of acute respiratory infections^30,31^. Viral infections in higher vertebrates elicit potent innate and adaptive host immune responses. However, an excessive or inappropriate immune response may also lead to a host pathology, which is often more severe than the direct effect of viral replication^32^. As a sequential response to the infection, lung inflammation is one of the important processes in pneumonia, since severe inflammation may cause lung failure and death. The suggested scenarios by which probiotics improve clinical symptoms and disease course include (1) inhibition of bacterial adhesion, (2) production of antimicrobial compounds, (3) enhanced mucosal barrier function, and (4) modulation of innate and adaptive immune cells^33,34^. This study demonstrated that probiotic co-administration may elicit an appropriate immune response to clear pathogens and reduce inflammation and tissue damage.

Previous studies have shown the complexity of differentiation of helper T cells and the influence of infection on Tregs, Th17 cells, and Th17 cell/Treg balance. In the case of *Mycoplasma* pneumonia, the ratio of Th17 cells/Tregs increased. On the other hand, another study demonstrated that a virus infection activates Tregs^35^. In influenza A virus infection characterized by lung inflammation, muramyl dipeptide significantly reduces both the viral load and lung inflammation as well as improves pulmonary function by increasing Tregs and diminished Th17 levels^36^. A previous study reported an increase in the Th17 cell/Treg ratio in patients with early ARDS, and a higher Th17 cell/Treg ratio was associated with a poorer prognosis, with a cutoff value of 0.79^21^. Consistent with this finding, we observed decreased Tregs and increased Th17 cell/Treg ratio in the early phase of pneumonia treatment, and probiotic administration facilitated Tregs and suppressed the Th17 cells/Treg ratio. The fraction of the Th17 cell population was maintained and Treg suppression was abolished by probiotic administration. Probiotics may strengthen the resilence returning to immune homeostasis which provide an appropriate response of the immune system.

Many studies have shown that the clinical impact and effectiveness of probiotics vary depending on the microbial species. We used the commercial probiotic product Medilac-DS, which is composed of *Bacillus subtilis* and *Enterococcus faecium*. These strains are widely used in clinical practice, and several clinical trials have been conducted on these strains previously. One study showed that therapy with the probiotic bacteria *B. subtilis* and *Enterococcus faecalis* is effective and safe for preventing VAP^13^. Based on clinical trial and meta-analysis results, Medilac-S was considered as part of standard care for ulcerative colitis in a Chinese population^37^. In a prospective study conducted in the Korean population showed that Medilac-DS is a safe and useful probiotic agent for the treatment of abdominal pain in patients with irritable bowel syndrome^38^.

This study has several strengths. First, this was a prospective clinical trial in which patients were randomly assigned and the clinical course was observed thereafter. Second, the demographic and baseline characteristics of the participants were homogeneous. No patient’s condition corresponded to the 2007 American Thoracic Society (ATS)/Infectious Diseases Society of America (IDSA) severe community-acquired pneumonia (CAP) criteria. Third, the patients enrolled had passed medical checks when they were recruited to the military, and thus did not have any comorbidities, and known risk factors for a poor prognosis of pneumonia, such as chronic heart, lung, liver, or renal disease; diabetes mellitus; alcoholism; malignancy; and asplenia, were not present. Fourth, the patients recruited were young healthy soldiers who were particular about maintaining their health, and therefore, inflammatory responses to the pathogen may be enhanced in these subjects.

This study also has some limitations. First, the number of participants enrolled in the study was relatively small. In addition, 27 participants did not attend a second follow-up, which may have influenced the results, since participants with good compliance or with persistent symptoms may have visited the hospital. Second, since the present study was an open-label study, some subjective factors may be influenced by the administration of an additional tablet. Third, all of the participants enrolled had non-severe pneumonia. The participants’ CURB-65 (acronym for Confusion, Urea, Respiratory rate, Blood pressure and age ≥ 65) scores were nearly 0 and their Pneumonia Severity Index (PSI) scores were very low, approximately 20, suggesting very low mortality rates. These patients usually did not need to be hospitalized and were administered intravenous antibiotics according to the guidelines. However, due to the specificity of the military setting, patients were hospitalized and received intravenous antibiotics. Moreover, most of the isolated organisms were those associated with atypical pneumonia, which may be because the participants lived in the military camp and were young (in their early 20 s). Fourth, statistically, clinical parameters and FACS results did not show between-group differences for the repeated measures (at enrollment, first follow-up, and second follow-up), this may be due to the fact that the results differed depending on the treatment and recovery phases. In addition, blood sampling for flow cytometric analysis was not performed for all the participants; only 4 to 8 samples were analyzed for each group due to the limitation of sample acquisition for analysis. Lastly, this study did not analyze the changes in intestinal or fecal microbiome composition with respect to probiotic administration.

Together, our data demonstrate improved clinical outcomes and reduced inflammation after co-administration of a probiotic with conventional antibiotic therapy. Probiotic administration may facilitate Tregs and suppress the Th17 cells/Treg ratio. These results support the beneficial effect of probiotics in the treatment of lung inflammation caused by pneumonia.

## Methods

### Subjects

An open-label, simple, randomized controlled trial was conducted among participants diagnosed with pneumonia at Naval Pohang Hospital, Pohang, South Korea, from May 2016 to January 2017. The diagnosis of pneumonia was based on the ATS/IDSA guidelines for CAP^25^. Suggestive clinical features, that is, a demonstrable infiltrate on chest radiography or computed tomography with or without supporting microbiological data, are required for the diagnosis of pneumonia. Individuals with HIV, syphilis, diabetes, hypertension, or other serious diseases, and steroid users were excluded. Young adults, aged 19 to 25, who met the above criteria and provided informed consent were included in the study. The number of participants was determined based on an effect size of 0.8, a two-sided alpha level of 0.05, and a statistical power of 80% using the statistical power analysis software G*Power (version 3.1) (G-Power Inc., German).Because we expected the participants to have similar baseline characteristics, 80 participants were enrolled and assigned to two groups in a 1:1 allocation ratio in a simple randomized manner.

### Study protocol

The total follow-up period was 2 weeks, and the participants were treated with intravenous antibiotics for the first 7 ± 2 days of the treatment period, followed by 7 ± 2 days of oral antibiotic therapy in the outpatient clinic. The first-line antibiotics were intravenous ceftriaxone and oral azithromycin during the first week followed by oral fluoroquinolone therapy for 1 week thereafter. The participants were assigned to two groups depending on the co-administration of the probiotic with antibiotic therapy. Patients were withdrawn from the study in instances in which the protocol was not properly followed, such as transfer to another hospital or exceeding the period of intravenous or oral antibiotic administration for the designated days. The primary outcome was to improve the clinical parameters of the participants: general condition, fever duration, serum hs-CRP levels, and abnormal stool patterns. The secondary outcome was the alteration of immune cells, in particular the distribution of Tregs and Th17 cells.

### Clinical parameters

The participants’ demographic characteristics and medical history were collected. We performed a systemic review of fever, chills, headache, dizziness, myalgia, cough, coryza, sputum, nausea, vomiting, constipation, diarrhea, chest pain, and abdominal pain. Vital signs were checked at baseline and during follow-ups. General condition was a subjective parameter in this study, and we collected related data using a visual analog scale (VAS). Participants reported their condition by stating their scores at weeks 1 and 2. Stool characteristics were assessed using the Bristol stool scale, with type 1 or 2 indicating constipation, type 3 to 5 indicating ideal stool, and type 6 or 7 indicating diarrhea. We monitored chest imaging findings and clinical symptoms during the follow-up period.

Hematological and biochemical laboratory parameters were assessed at baseline and during follow-ups. Furthermore, sputum and blood cultures were performed. Microbial analyses, i.e., respiratory PCR for adenovirus, RSV, influenza, parainfluenza, bocavirus, metapneumovirus, coronavirus, rhinovirus; pneumococcus urinary antigen test; and *Mycoplasma* IgM test were also performed.

### Flow cytometry

Approximately 10 cc of venous blood was collected from the participants at baseline, and at weeks 1 and 2. Peripheral blood mononuclear cells were isolated using Lymphoprep™ (STEMCELL Technologies Inc., Canada) following the manufacturer’ s instructions. Flow cytometric analysis was performed using an LSR II flow cytometer (Becton, Dickinson and Company Inc., USA) for CD4, CD25, Foxp3, and RORγt, markers for Tregs and Th17 cells, respectively. The specific cell populations were analyzed at baseline, and at weeks 1 and 2.

### Probiotic administration

In the probiotic group, a probiotic named Medilac-DS enteric-coated capsules (Hanmi Pharma Inc., South Korea), which contains a total of one billion live *B. subtilis* and *Streptococcus faecium* strains at a ratio of 1:9, was administered. The capsules (250 mg each) were administered orally three times a day. A probiotic was co-administered with intravenous or oral antibiotics for 14 ± 4 days of the treatment period.

### Statistical analysis

Regarding the intent-to-treat analysis concept, data from all subjects were analyzed. Student’s *t*-test, paired *t*-test, and chi-square test were used for the analysis of demographic and clinical variables. The *Δ*-value, the difference between the levels of the follow-up and the baseline, was used to compare changes in the parameters between groups. Regarding the per-protocol analysis concept, the within-group and between-group differences in the changes from the baseline to the repeated measures (at weeks 1 and 2) were assessed by mixed ANOVA with post-hoc comparisons. Graphical images were expressed as the mean ± standard error of the mean (SEM) values by using GraphPad Prism software (version 5.0; GraphPad Software, La Jolla, CA). All statistical analyses were conducted using IBM SPSS software 21.0 (SPSS Inc., Chicago, IL, USA), and P-values were considered significant at < 0.05. All statistical tests were two-tailed.

### Ethics statement

This study protocol was consistent with the ethical guidelines of the 1975 Declaration of Helsinki and was approved by the institutional review board of the Korean Military Medical Research Project (Approval No. AFMC-16033-IRB-16-026). Written informed consent was obtained from all study participants. This trial was registered at Clinical Research Information Service 09/08/2020―registration no. KCT0005411 (date of first registration, 06/27/2016).

## Supplementary information

Supplementary Information.
