# Supplementary material for: Addition of probiotics to antibiotics improves the clinical course of pneumonia in young people without comorbidities: a randomized controlled trial
Source: Sci Rep. 2021 Jan 13;11:926. doi: 10.1038/s41598-020-79630-2 (PMC7806890; doi:10.1038/s41598-020-79630-2)
Supplement: Supplementary file 1 — Supplementary Information. [file 41598_2020_79630_MOESM1_ESM.docx]

**Addition of probiotics to antibiotics improves the clinical course of pneumonia in young people without comorbidities: a randomized controlled trial**

Chang Hun Lee^1,2^, Yun Jung Choi^3^, Seung Young Seo^2^, Seong Hun Kim^2^, In Hee Kim^2^, Sang Wook Kim^2^, Soo Teik Lee^2^, and Seung Ok Lee^2,*^

^1^Department of Internal Medicine, Naval Pohang Hospital, Pohang, South Korea

^2^Division of Gastroenterology, Department of Internal Medicine, Jeonbuk National University Medical School, Research Institute of Clinical Medicine of Jeonbuk National University-Biomedical Research Institute of Jeonbuk National University Hospital, Jeonju, South Korea

^3^Division of Rheumatology, Department of Internal Medicine, Jeonbuk National University Medical School, Research Institute of Clinical Medicine of Jeonbuk National University-Biomedical Research Institute of Jeonbuk National University Hospital, Jeonju, South Korea

Address correspondence to:

Seung Ok Lee, MD, PhD. Department of Internal Medicine, Jeonbuk National University Medical School, Research Institute of Clinical Medicine of Jeonbuk National University-Biomedical Research Institute of Jeonbuk National University Hospital, 20 Geonjiro, Dukjingu, Jeonju, Jeonbuk, 54907, South Korea. Phone: 82-63-250-1289; E-mail: [solee@jbnu.ac.kr](mailto:solee@jbnu.ac.kr)

**Contents**

1. Supplementary Figure

2. Supplementary Tables

**1. Supplementary Figure**


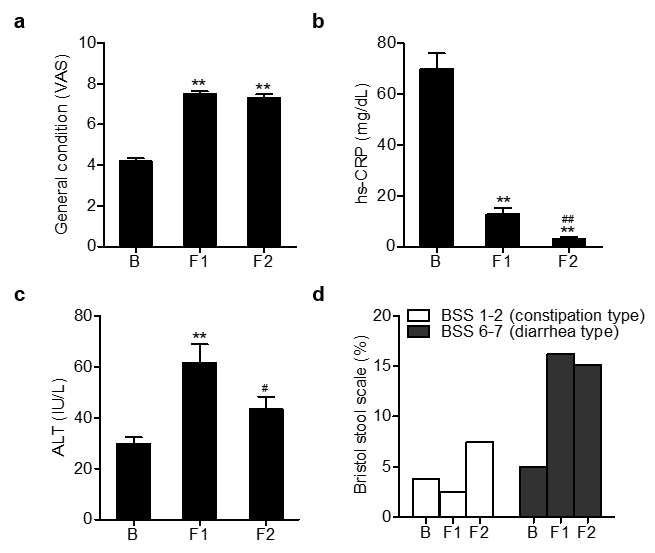


**Figure S1. Changes of clinical and biochemical parameters during the study enrollment.** Values are mean ± SEM. **, P < 0.01 versus baseline, ^##^, P < 0.05, and ^##^, P < 0.01 versus F1. baseline; F1, 1 week after treatment; F2, 2 weeks after treatment.


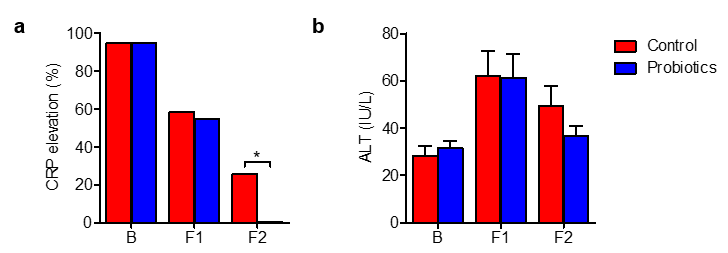


**Figure S2. Comparison of clinical manifestations depending on the administration of the probiotic.** Values are mean ± SEM. *, P < 0.05. B, baseline; F1, 1 week after treatment; F2, 2 weeks after treatment.

**2. Supplementary Tables**

**Table S1. Microbiologic data as a diagnostic test for etiology of pneumonia**

| **Characteristics** | **Control group**  **(n=40)** | **Probiotic group**  **(n=40)** | **P value** |
| --- | --- | --- | --- |
| Sputum AFB + | 0 (0.0%) | 0 (0.0%) | 1.000 |
| Sputum culture + | 0 (0.0%) | 2 (5.0%) | 0.474 |
| Blood culture + | 1 (2.5%) | 0 (0.0%) | 1.000 |
| Pneumococcus urinary antigen | 0 (0.0%) | 1 (2.6%) | 0.990 |
| Mycoplasma | 14 (35.0%) | 18 (47.4%) | 0.379 |
| Respiratory virus PCR + | 23 (57.5%) | 24 (60.0%) | 1.000 |
| Adenovirus | 13 (32.5%) | 17 (42.5%) | 0.488 |
| RSV | 0 (0.0%) | 1 (2.5%) | 1.000 |
| Influenza | 0 (0.0%) | 1 (2.5%) | 1.000 |
| Parainfluenza | 1 (2.5%) | 0 (0.0%) | 1.000 |
| Bocavirus | 0 (0.0%) | 0 (0.0%) | - |
| Metapneumovirus | 0 (0.0%) | 0 (0.0%) | - |
| Coronavirus | 0 (0.0%) | 0 (0.0%) | - |
| Rhinovirus | 14 (35.0%) | 16 (40.0%) | 0.817 |

AFB, acid fast bacillus; PCR, polymerase chain reaction; RSV, respiratory syncytial virus.

**Table S2. Clinical and laboratory parameters 1 week after treatment**

| **Characteristics** | **Control group**  **(n=40)** | **Probiotic group**  **(n=40)** | **Overall**  **(n=80)** | **P value** |
| --- | --- | --- | --- | --- |
| **General condition (VAS)** | **7.2 ± 1.2** | **7.8 ± 1.1** | **7.5 ± 1.2** | **0.016** |
| Clinical manifestations |  |  |  |  |
| Fever | 0 (0.0%) | 0 (0.0%) | 0 (0.0%) | - |
| Chills | 1 (2.5%) | 0 (0.0%) | 1 (1.2%) | 1.000 |
| Headache | 38 (95.0%) | 39 (97.5%) | 77 (96.2%) | 1.000 |
| Myalgia | 1 (2.5%) | 0 (0.0%) | 1 (1.2%) | 1.000 |
| Cough | 19 (47.5%) | 24 (60.0%) | 43 (53.8%) | 0.370 |
| Coryza | 6 (15.0%) | 6 (15.0%) | 12 (15.0%) | 1.000 |
| Sputum | 19 (47.5%) | 16 (40.0%) | 35 (43.8%) | 0.652 |
| Sore throat | 0 (0.0%) | 0 (0.0%) | 0 (0.0%) | - |
| Chest pain | 4 (10.0%) | 4 (10.0%) | 8 (10.0%) | 1.000 |
| Abdominal pain | 1 (2.5%) | 0 (0.0%) | 1 (1.2%) | 1.000 |
| Bristol Stool Scale |  |  |  | 0.215 |
| Type 1 or 2 | 2 (5.0%) | 0 (0.0%) | 2 (2.5%) |  |
| Type 3, 4, or 5 | 30 (75.0%) | 35 (87.5%) | 65 (81.2%) |  |
| Type 6 or 7 | 8 (20.0%) | 5 (12.5%) | 13 (16.2%) |  |
| Vital Sign |  |  |  |  |
| Systolic BP, mmHg | 117.9 ± 10.9 | 116.9 ± 10.0 | 117.4 ± 10.4 | 0.670 |
| Diastolic BP, mmHg | 73.2 ± 8.9 | 74.3 ± 9.2 | 73.8 ± 9.0 | 0.596 |
| **HR, beat per min** | **65.7 ± 8.4** | **69.9 ± 8.5** | **67.8 ± 8.7** | **0.029** |
| RR, breath per min |  |  |  |  |
| Body temperature, ℃ | 36.3 ± 0.2 | 35.5 ± 5.2 | 35.9 ± 3.7 | 0.312 |
| Fever subsidence | 25 (100.0%) | 26 (100.0%) | 51 (100.0%) | 1.000 |
| **Fever duration (days)** | **3.3 ± 1.3** | **2.3 ± 0.7** | **2.8 ± 1.1** | **0.003** |
| WBC, /μl | 5445.8 ± 1978.8 | 5840.8 ± 1567.7 | 5643.2 ± 1784.9 | 0.325 |
| Hemoglobin, g/dl | 14.4 ± 1.1 | 14.1 ± 1.3 | 14.3 ± 1.2 | 0.254 |
| Platelet, /μl | 271.1 ± 80.1 | 317.3 ± 206.5 | 294.2 ± 157.3 | 0.193 |
| PT (INR) | 1.2 ± 0.1 | 1.1 ± NA | 1.2 ± 0.1 |  |
| hs-CRP, mg/dl | 13.7 ± 21.5 | 11.5 ± 23.9 | 12.6 ± 22.6 | 0.662 |
| CRP elevation | 23 (57.5%) | 22 (55.0%) | 45 (56.2%) | 1.000 |
| ESR, mm/hr | 14.8 ± 11.2 | 6.2 ± 3.6 | 10.5 ± 9.0 | 0.140 |
| ALP, IU/l | 73.3 ± 62.8 | 60.7 ± 15.5 | 66.9 ± 45.6 | 0.231 |
| AST, IU/l | 41.8 ± 34.1 | 34.2 ± 17.2 | 38.0 ± 27.1 | 0.215 |
| AST elevation | 12 (30.0%) | 10 (25.0%) | 22 (27.5%) | 0.802 |
| ALT, IU/l | 62.2 ± 65.0 | 61.4 ± 63.4 | 61.8 ± 63.8 | 0.953 |
| ALT elevation | 20 (50.0%) | 22 (55.0%) | 42 (52.5%) | 0.823 |
| Total bilirubin, mg/dl | 0.4 ± 0.2 | 0.4 ± 0.2 | 0.4 ± 0.2 | 0.644 |
| Protein, g/dl | 6.5 ± 0.5 | 6.4 ± 0.4 | 6.5 ± 0.5 | 0.296 |
| Albumin, g/dl | 3.9 ± 0.3 | 3.8 ± 0.3 | 3.8 ± 0.3 | 0.598 |
| BUN, mg/dl | 10.4 ± 2.3 | 9.6 ± 2.7 | 10.0 ± 2.5 | 0.163 |
| Creatinine, mg/dl | 0.8 ± 0.2 | 0.8 ± 0.1 | 0.8 ± 0.1 | 0.225 |
| Na, mmol/l | 136.8 ± 2.4 | 137.1 ± 3.2 | 137.0 ± 2.8 | 0.652 |
| K, mmol/l | 4.5 ± 0.3 | 4.4 ± 0.4 | 4.4 ± 0.3 | 0.604 |
| Cl, mmol/l | 101.9 ± 2.3 | 102.8 ± 2.5 | 102.3 ± 2.5 | 0.083 |
| LD, IU/l | 185.4 ± 41.6 | 172.5 ± 42.7 | 178.9 ± 42.4 | 0.194 |

**Table S3. Clinical and laboratory parameters 2 weeks after treatment**

| **Characteristics** | **Control group**  **(n=29)** | **Probiotic group**  **(n=24)** | **Overall**  **(n=53)** | **P value** |
| --- | --- | --- | --- | --- |
| **General condition (VAS)** | **6.9 ± 1.4** | **7.8 ± 1.0** | **7.3 ± 1.3** | **0.007** |
| Clinical manifestations |  |  |  |  |
| Fever | 0 (0.0%) | 0 (0.0%) | 0 (0.0%) | - |
| Chills | 0 (0.0%) | 0 (0.0%) | 0 (0.0%) | - |
| Headache | 2 (6.9%) | 1 (4.2%) | 3 (5.7%) | 1.000 |
| Myalgia | 0 (0.0%) | 0 (0.0%) | 0 (0.0%) | - |
| Cough | 15 (51.7%) | 9 (37.5%) | 24 (45.3%) | 0.448 |
| Coryza | 13 (44.8%) | 6 (25.0%) | 19 (35.8%) | 0.226 |
| Sputum | 13 (44.8%) | 4 (16.7%) | 17 (32.1%) | 0.059 |
| Sore throat | 0 (0.0%) | 0 (0.0%) | 0 (0.0%) | - |
| Chest pain | 3 (10.3%) | 1 (4.2%) | 4 (7.5%) | 0.745 |
| Abdominal pain | 1 (3.4%) | 0 (0.0%) | 1 (1.9%) | 1.000 |
| **Bristol Stool Scale** |  |  |  | **0.013** |
| Type 1 or 2 | 4 (13.8%) | 0 (0.0%) | 4 (7.5%) |  |
| Type 3, 4, or 5 | 18 (62.1%) | 23 (95.8%) | 41 (77.4%) |  |
| Type 6 or 7 | 7 (24.1%) | 1 (4.2%) | 8 (15.1%) |  |
| Vital Sign |  |  |  |  |
| Systolic BP, mmHg | 123.1 ± 11.1 | 120.0 ± 13.6 | 121.7 ± 12.3 | 0.389 |
| Diastolic BP, mmHg | 72.2 ± 10.2 | 69.9 ± 9.3 | 71.2 ± 9.8 | 0.408 |
| HR, beat per min | 67.9 ± 10.5 | 66.5 ± 7.7 | 67.2 ± 9.3 | 0.606 |
| RR, breath per min | 16.7 ± 2.1 | 16.7 ± 4.8 | 16.7 ± 3.5 | 0.946 |
| Body temperature, ℃ | 36.4 ± 0.3 | 35.5 ± 3.5 | 36.0 ± 2.4 | 0.238 |
| WBC, /μl | 5499.6 ± 1235.1 | 5828.8 ± 1528.2 | 5654.5 ± 1376.6 | 0.400 |
| Hemoglobin, g/dl | 14.0 ± 1.1 | 14.1 ± 1.3 | 14.0 ± 1.2 | 0.683 |
| Platelet, /μl | 285.3 ± 64.8 | 312.1 ± 77.2 | 297.9 ± 71.5 | 0.184 |
| **hs-CRP, mg/dl** | **4.5 ± 6.5** | **1.2 ± 0.8** | **3.0 ± 5.0** | **0.015** |
| **CRP elevation** | **7 (25.9%)** | **0 (0.0%)** | **7 (13.7%)** | **0.023** |
| ALP, IU/l | 69.1 ± 19.7 | 68.9 ± 15.5 | 69.0 ± 17.7 | 0.957 |
| AST, IU/l | 33.0 ± 20.8 | 25.6 ± 6.2 | 29.5 ± 16.0 | 0.089 |
| AST elevation | 5 (18.5%) | 1 (4.2%) | 6 (11.8%) | 0.249 |
| ALT, IU/l | 49.5 ± 43.6 | 36.5 ± 21.5 | 43.4 ± 35.3 | 0.178 |
| ALT elevation | 11 (40.7%) | 9 (37.5%) | 20 (39.2%) | 1.000 |
| Total bilirubin, mg/dl | 0.7 ± 0.3 | 0.8 ± 0.4 | 0.8 ± 0.3 | 0.088 |
| Protein, g/dl | 6.8 ± 1.4 | 7.0 ± 0.4 | 6.9 ± 1.1 | 0.443 |
| Albumin, g/dl | 4.4 ± 0.6 | 4.4 ± 0.3 | 4.4 ± 0.5 | 0.954 |
| BUN, mg/dl | 12.3 ± 2.6 | 12.0 ± 2.8 | 12.2 ± 2.7 | 0.663 |
| Creatinine, mg/dl | 1.5 ± 3.0 | 0.9 ± 0.2 | 1.2 ± 2.2 | 0.281 |
| Na, mmol/l | 137.9 ± 1.6 | 138.0 ± 1.7 | 138.0 ± 1.7 | 0.787 |
| **K, mmol/l** | **4.1 ± 0.2** | **4.3 ± 0.3** | **4.2 ± 0.3** | **0.010** |
| Cl, mmol/l | 101.8 ± 1.5 | 102.0 ± 1.9 | 101.9 ± 1.7 | 0.658 |
| **LD, IU/l** | **198.4 ± 56.3** | **162.6 ± 27.9** | **180.9 ± 47.8** | **0.010** |
